# Supplementary figures and images for: Roles for mycobacterial DinB2 in frameshift and substitution mutagenesis
Source: eLife. 2023 May 4;12:e83094. doi: 10.7554/eLife.83094 (PMC10159617; doi:10.7554/eLife.83094)

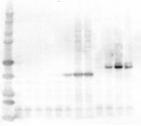

Supplement: Figure 1—source data 1. [file elife-83094-fig1-data1.zip › Fig 1/1A/Fig1A no labels.tif]

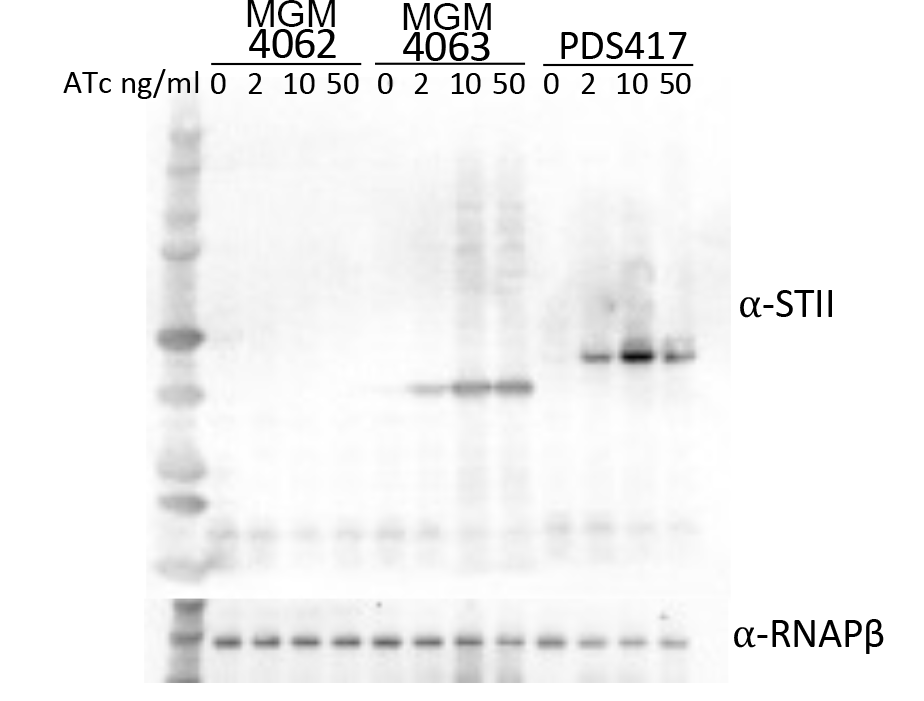

Supplement: Figure 1—source data 1. [file elife-83094-fig1-data1.zip › Fig 1/1A/Fig1A labels.tif]

vector  
DinB2-STII  
D107A  
L14F

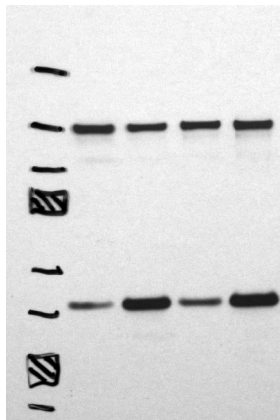

RpoB

RecA

Supplement: Figure 1—source data 1. [file elife-83094-fig1-data1.zip › Fig 1/1G/Fig IG label.pdf]

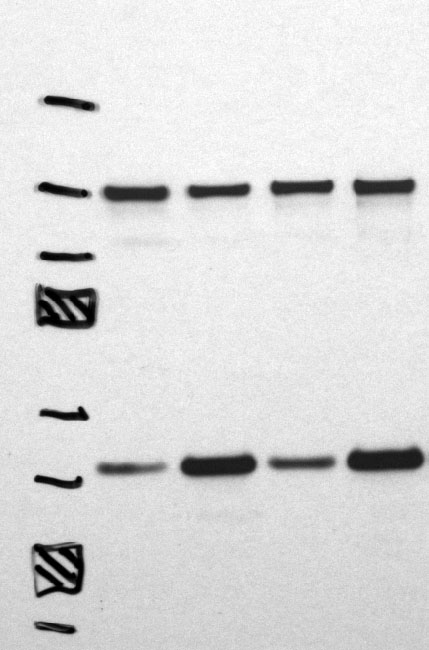

Supplement: Figure 1—source data 1. [file elife-83094-fig1-data1.zip › Fig 1/1G/Fig IG no label.jpg]

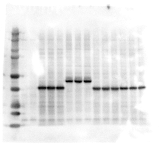

Supplement: Figure 1—source data 1. [file elife-83094-fig1-data1.zip › Fig 1/1E/Fig 1E no labels.tif]

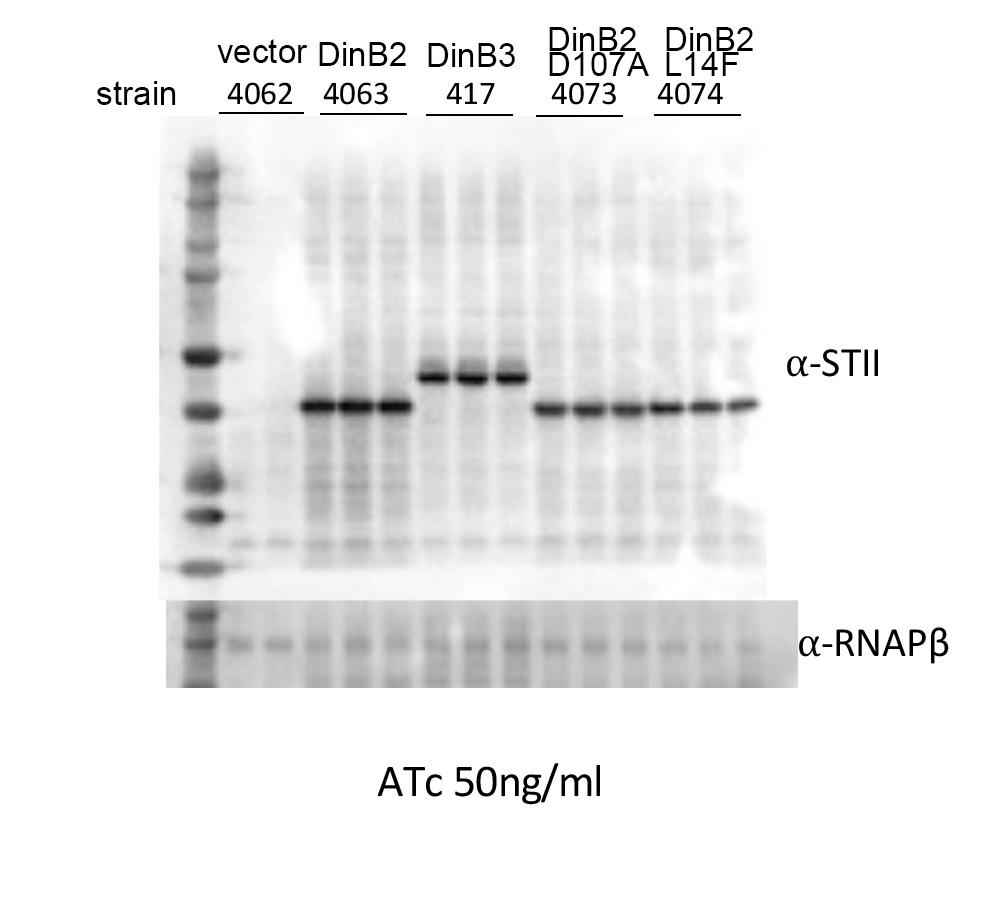

Supplement: Figure 1—source data 1. [file elife-83094-fig1-data1.zip › Fig 1/1E/Fig 1E labels.tif]

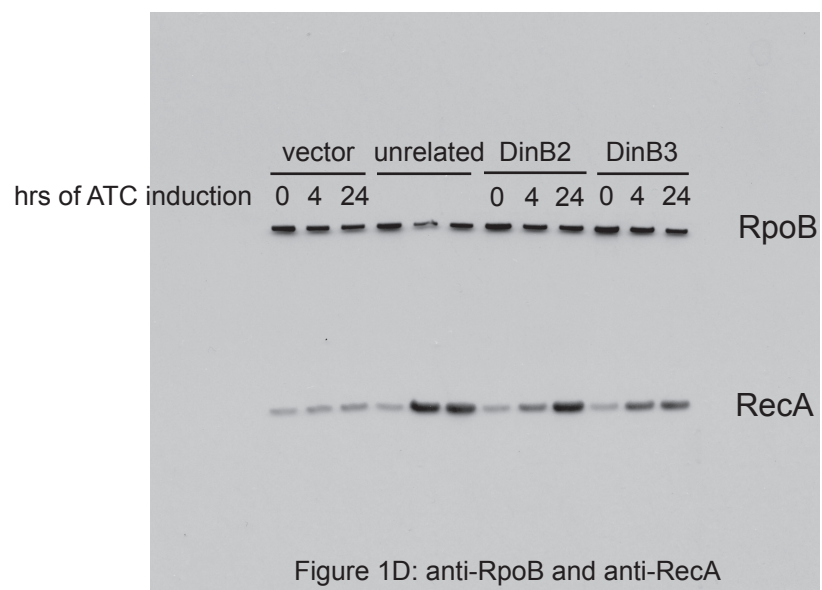

Supplement: Figure 1—source data 1. [file elife-83094-fig1-data1.zip › Fig 1/1D/Fig 1D labels.pdf]

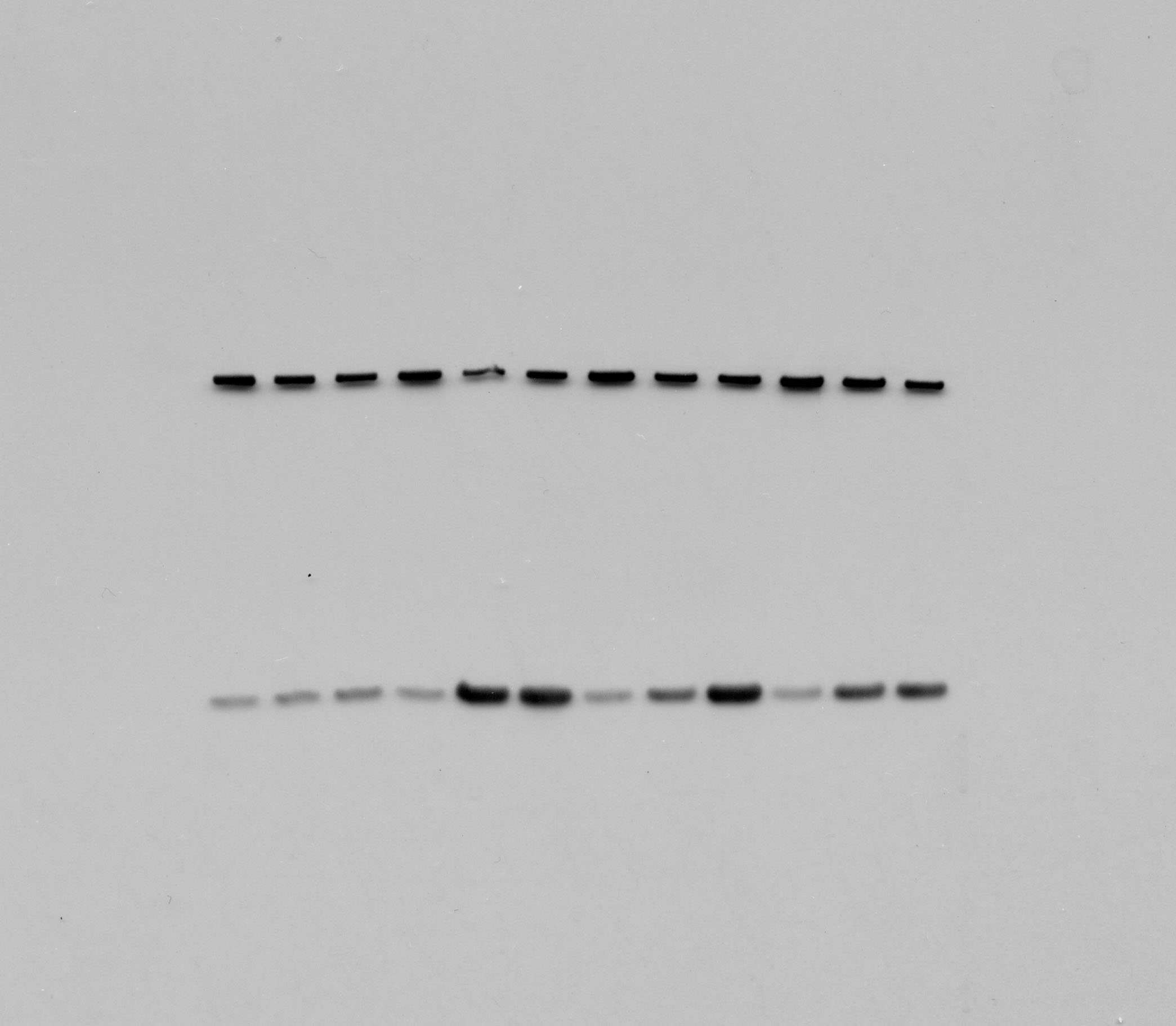

Supplement: Figure 1—source data 1. [file elife-83094-fig1-data1.zip › Fig 1/1D/Fig 1D no labels.jpg]

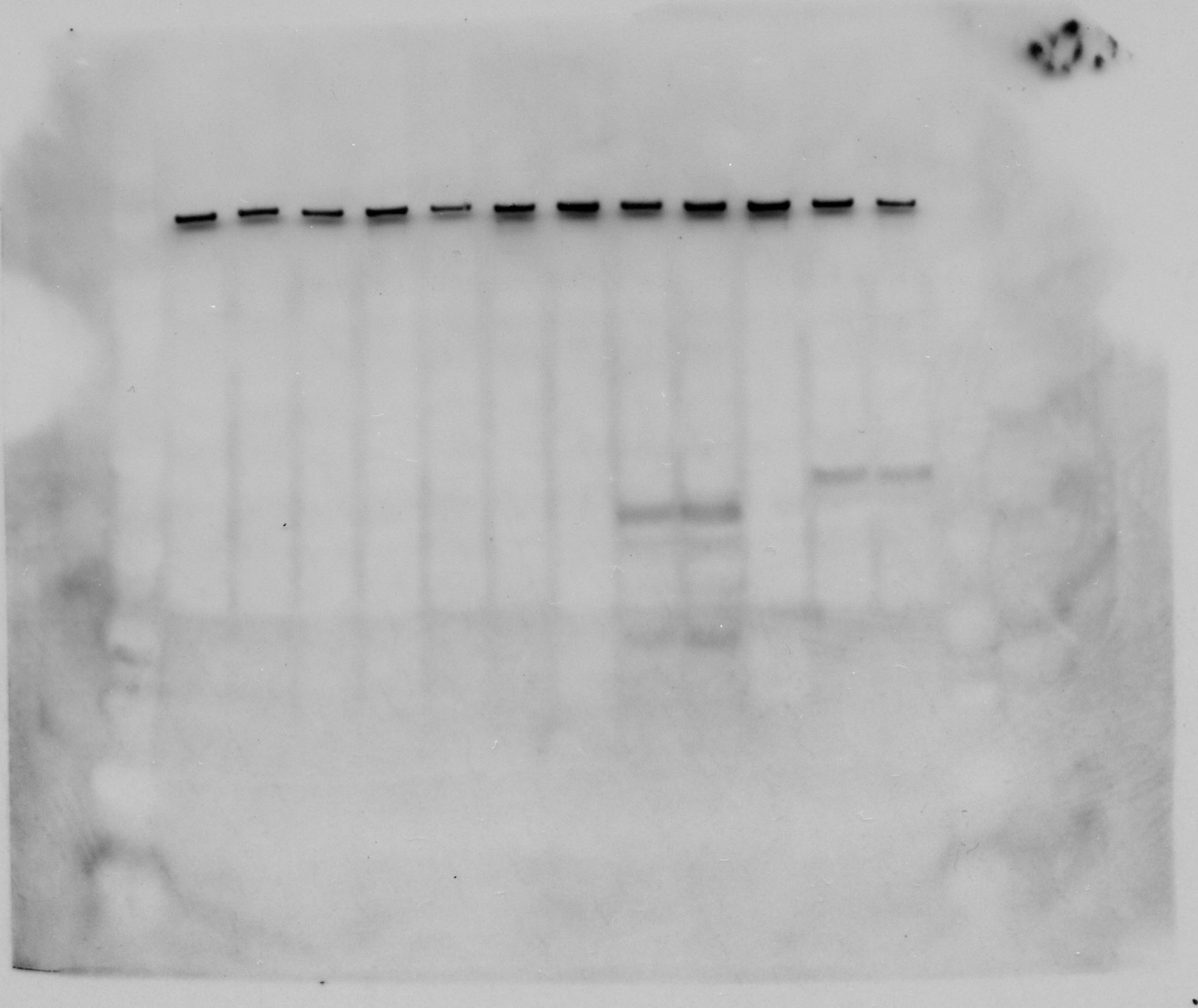

Supplement: Figure 1—figure supplement 1—source data 1. [file elife-83094-fig1-figsupp1-data1.zip › Fig1-fig supp 1 source gel data/Fig 1A source data no labels.jpg]

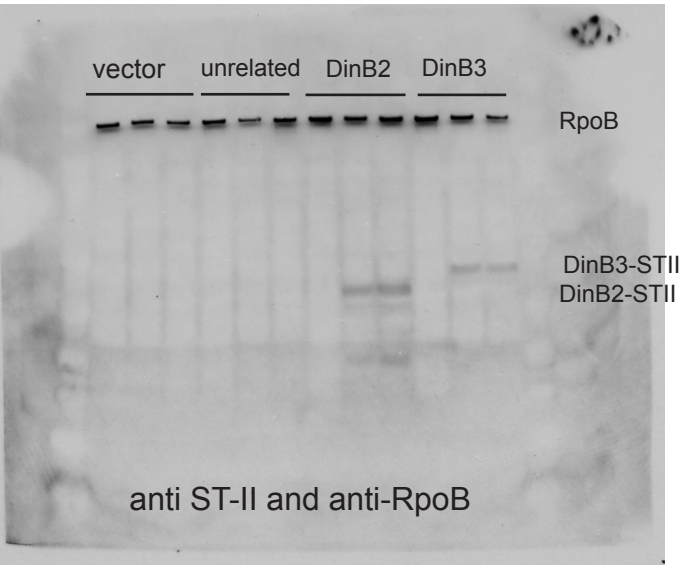

Supplement: Figure 1—figure supplement 1—source data 1. [file elife-83094-fig1-figsupp1-data1.zip › Fig1-fig supp 1 source gel data/1A western annotated.pdf]

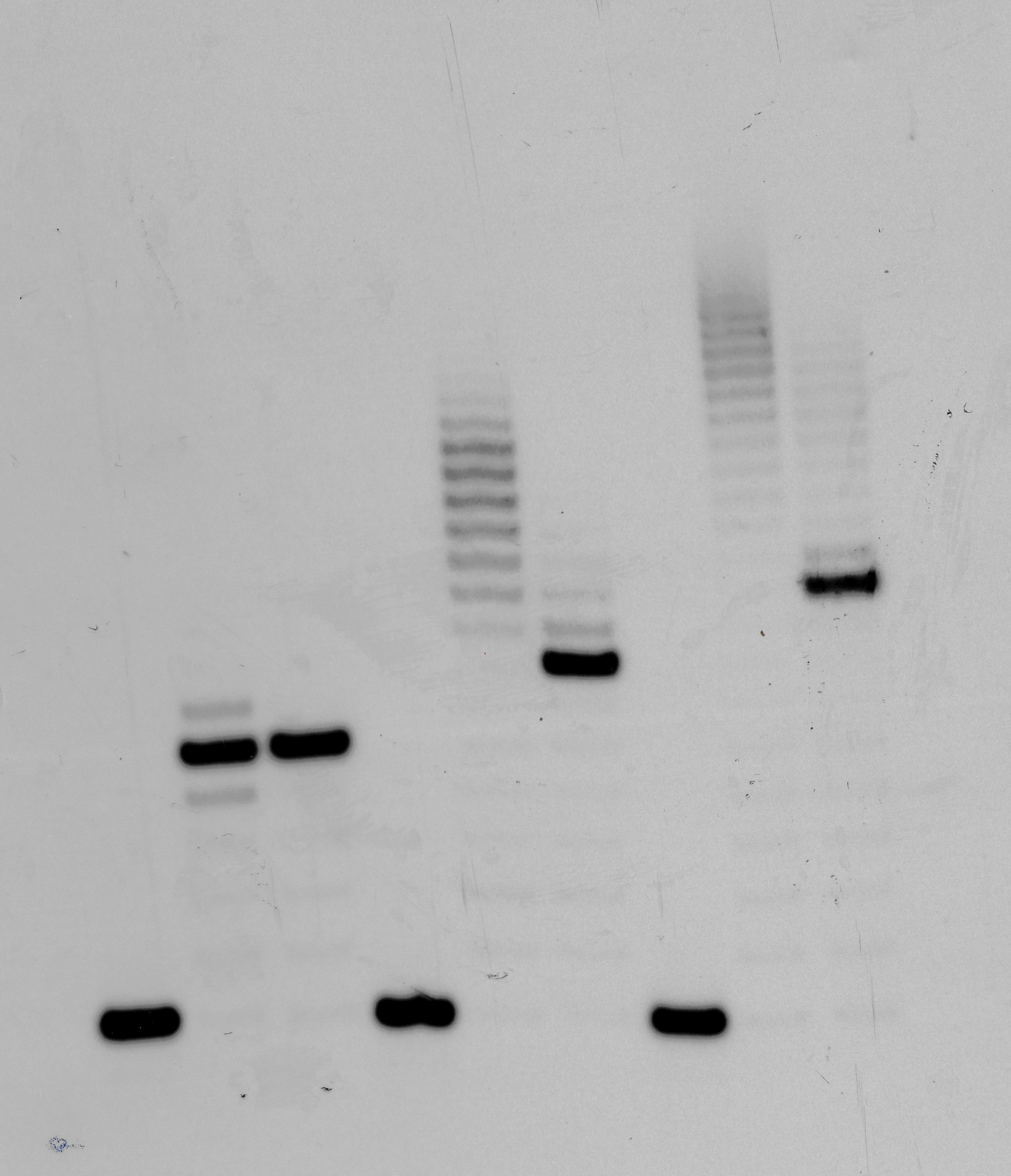

Supplement: Figure 3—source data 1. [file elife-83094-fig3-data1.zip › Fig 3/3A source file/Figure 3A source file no label.tiff]

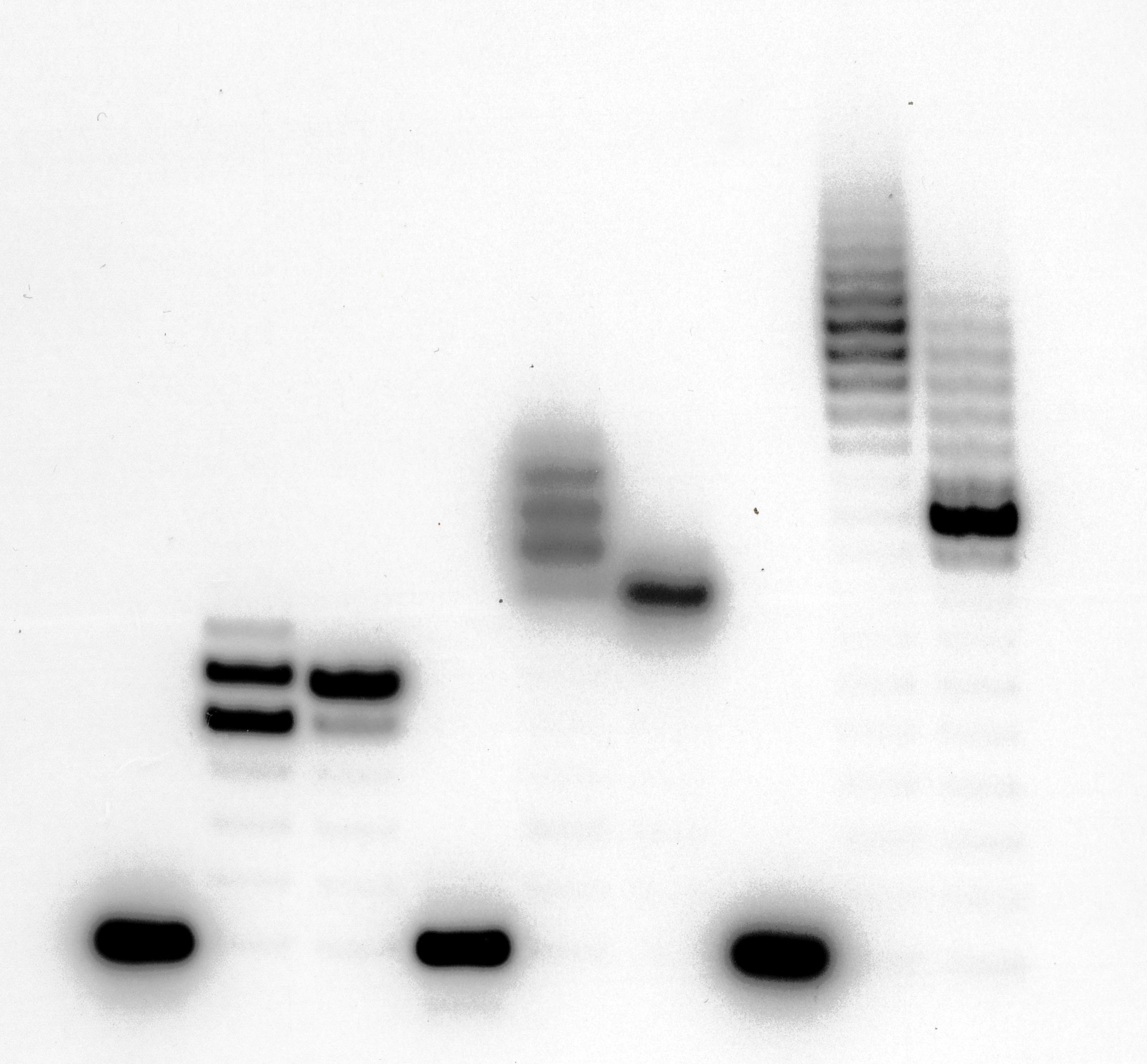

Supplement: Figure 3—source data 1. [file elife-83094-fig3-data1.zip › Fig 3/3B source file/Fig 3B no label.tiff]

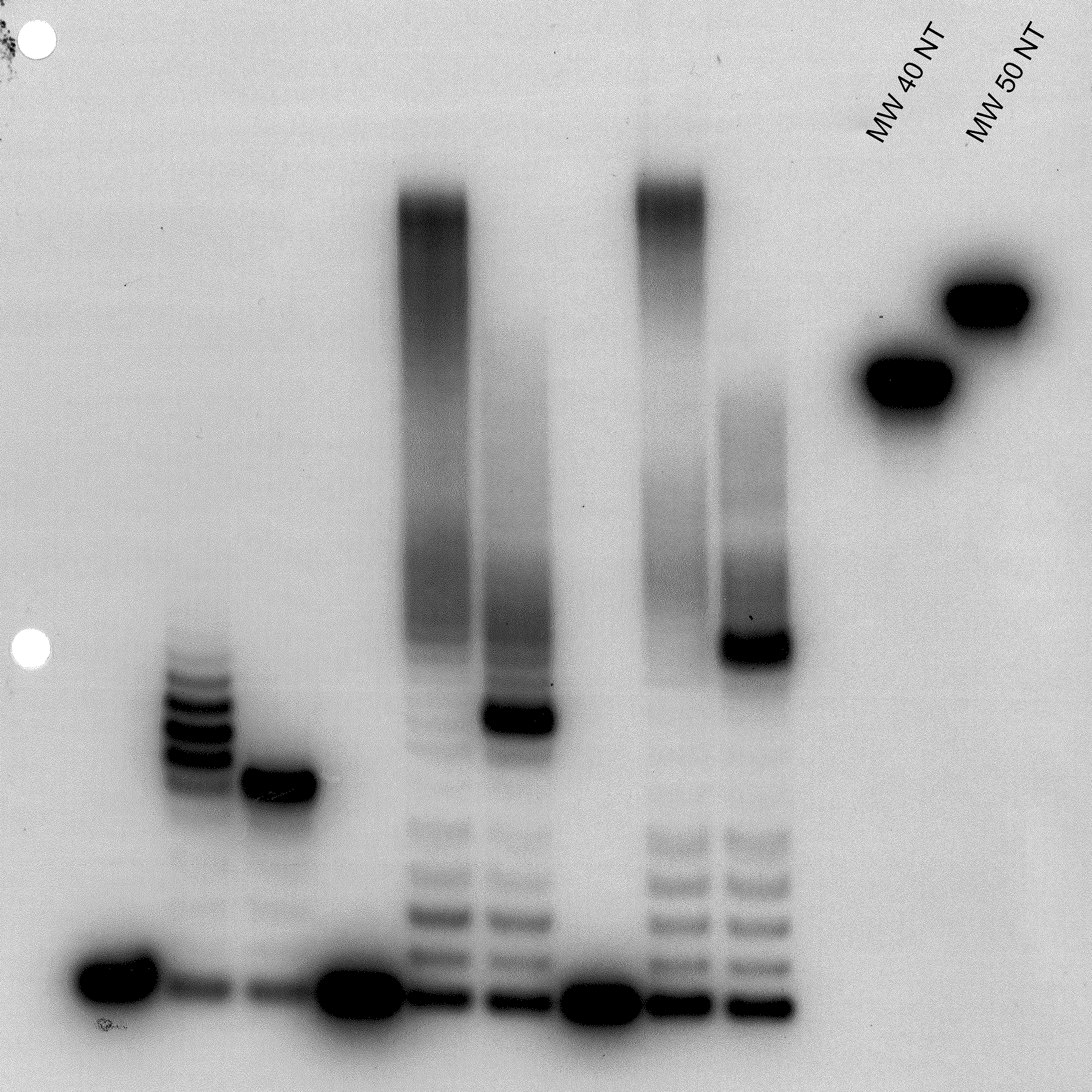

Supplement: Figure 4—source data 1. [file elife-83094-fig4-data1.zip › Fig 4/4B/Fig 4B label.tif]

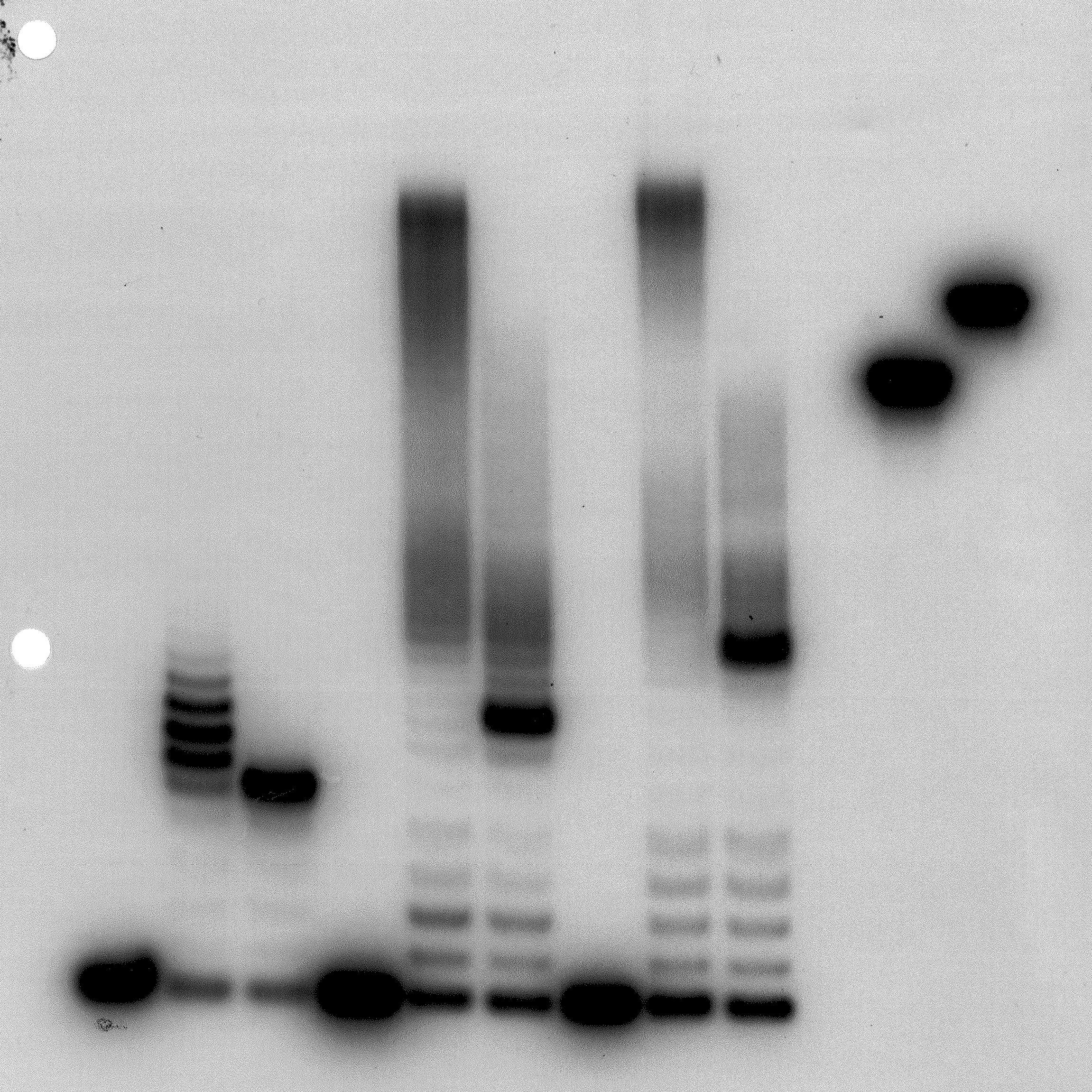

Supplement: Figure 4—source data 1. [file elife-83094-fig4-data1.zip › Fig 4/4B/Fig 4B no label.tiff]

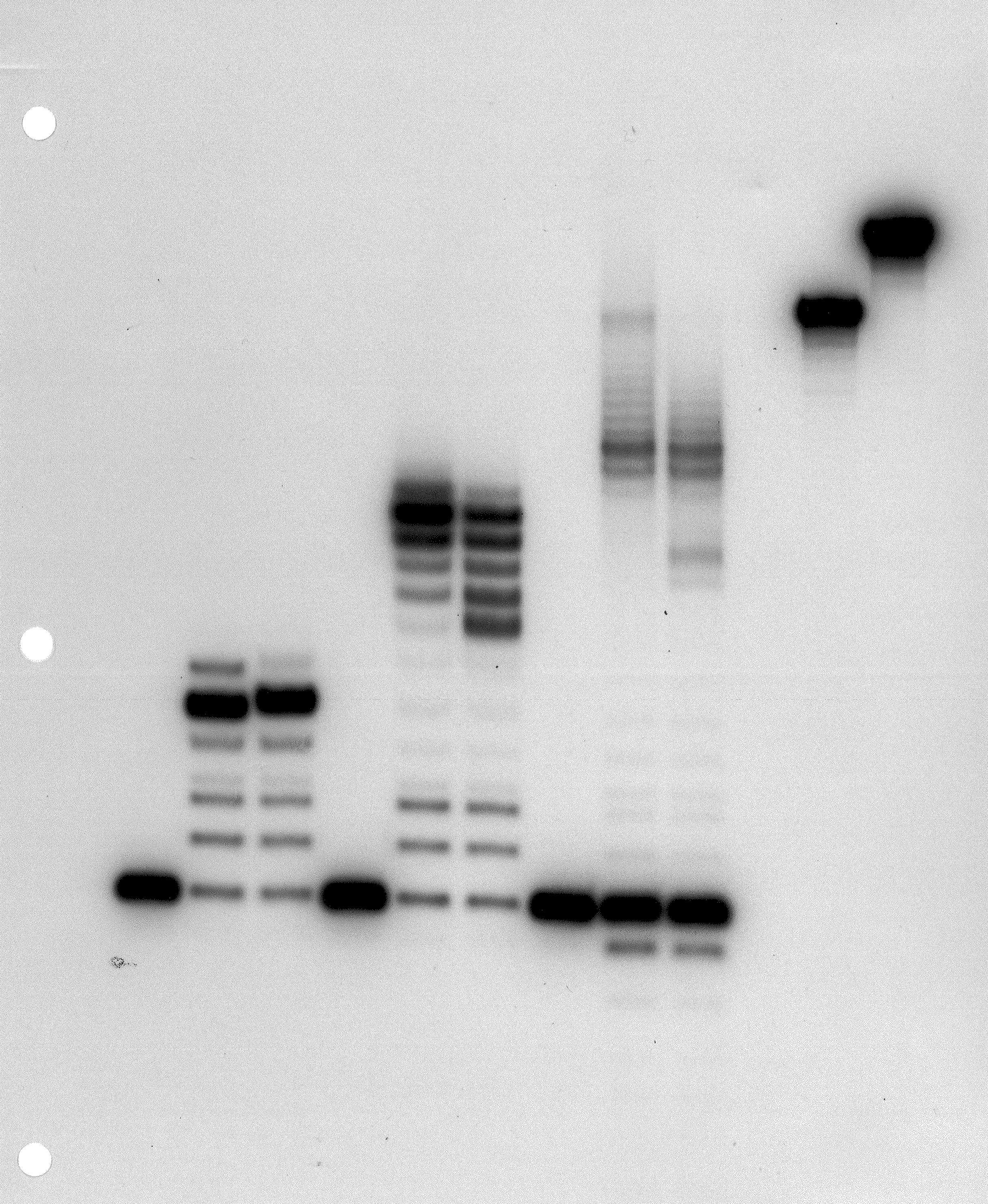

Supplement: Figure 4—source data 1. [file elife-83094-fig4-data1.zip › Fig 4/4A/Fig 4A no label.tiff]

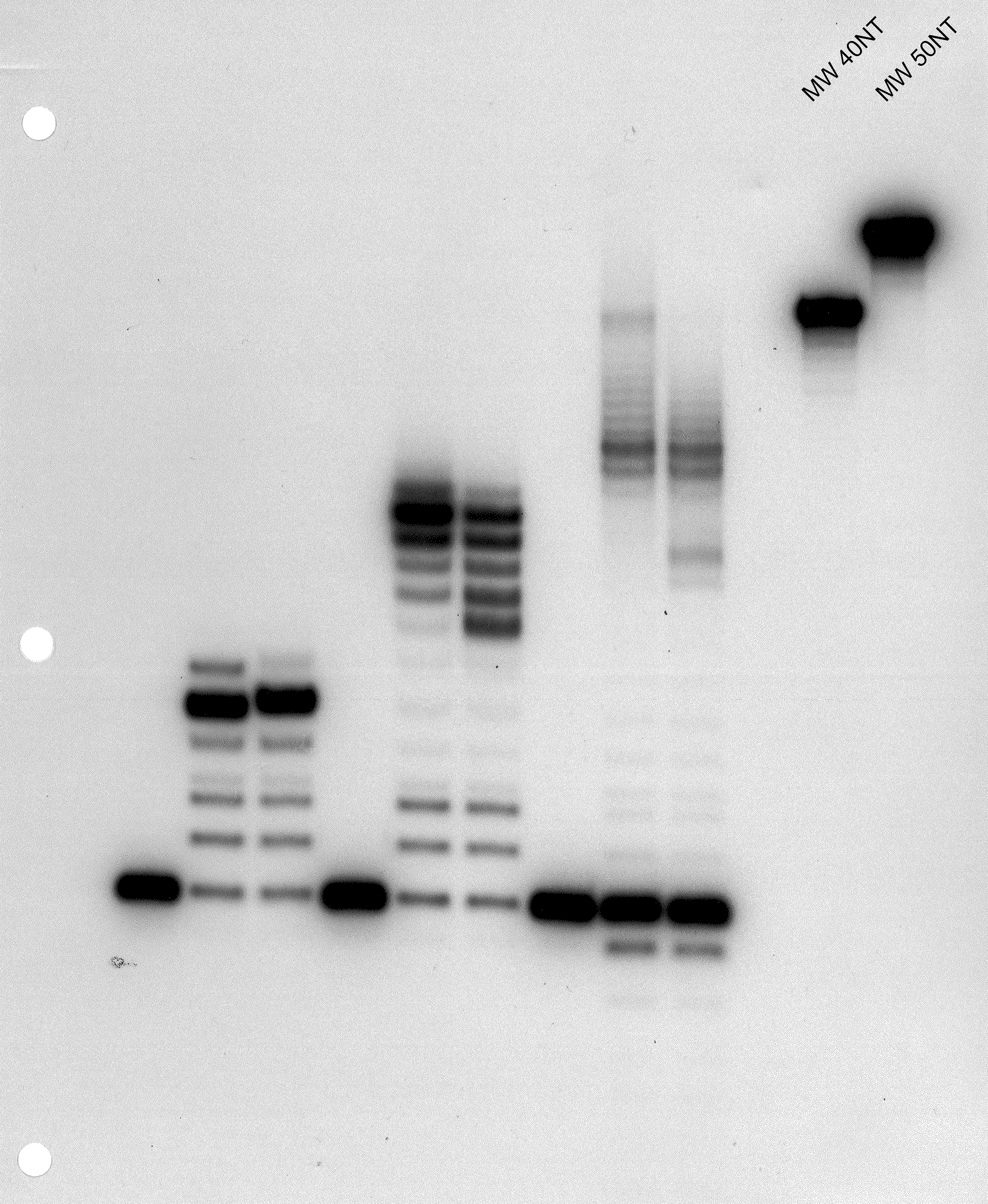

Supplement: Figure 4—source data 1. [file elife-83094-fig4-data1.zip › Fig 4/4A/Fig 4A labels.tif]

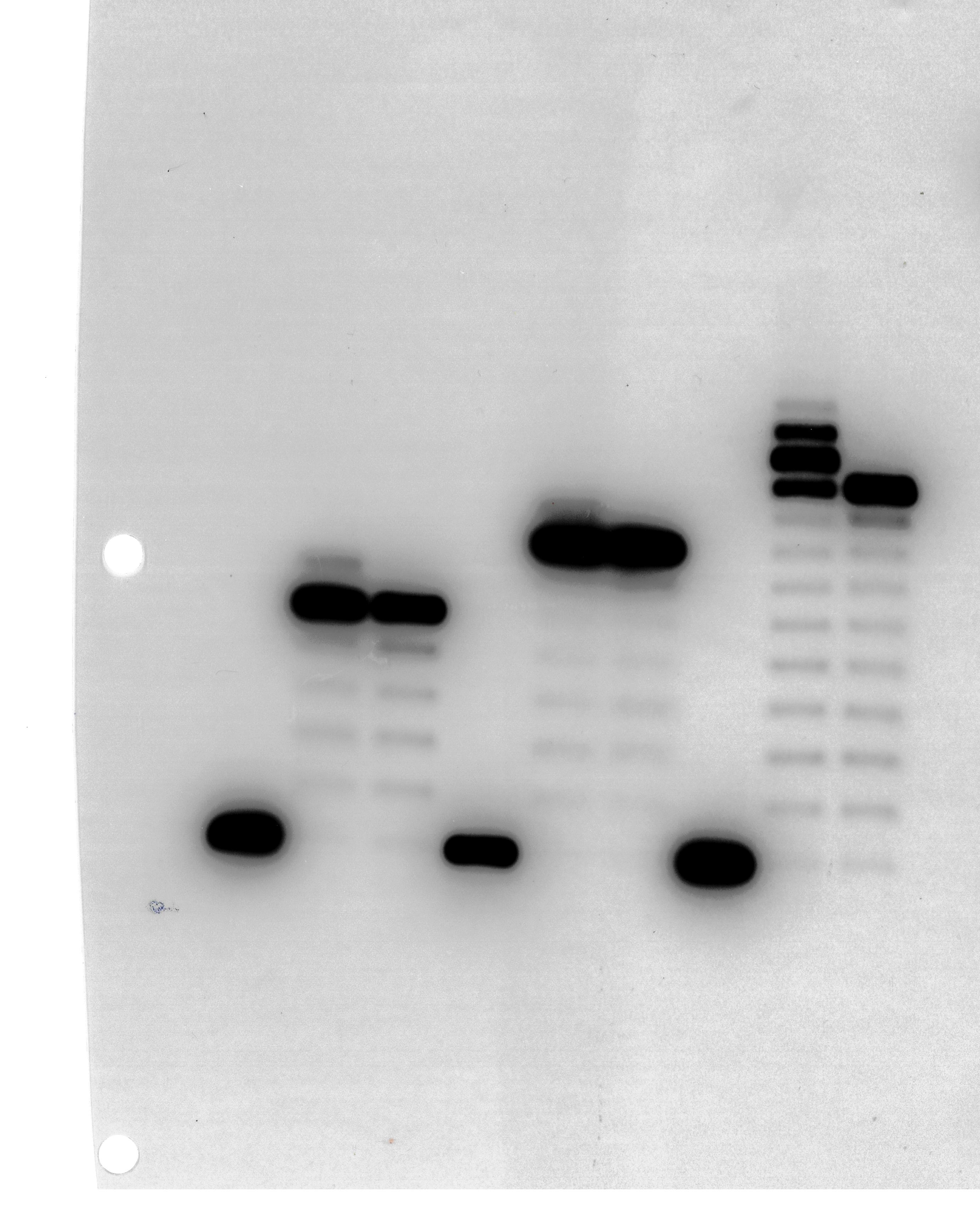

Supplement: Figure 4—figure supplement 1—source data 1. [file elife-83094-fig4-figsupp1-data1.zip › Fig4-fig supp 1/Fig4-fig supp 1A/Fig 4-fig supp 1A no label.tif]

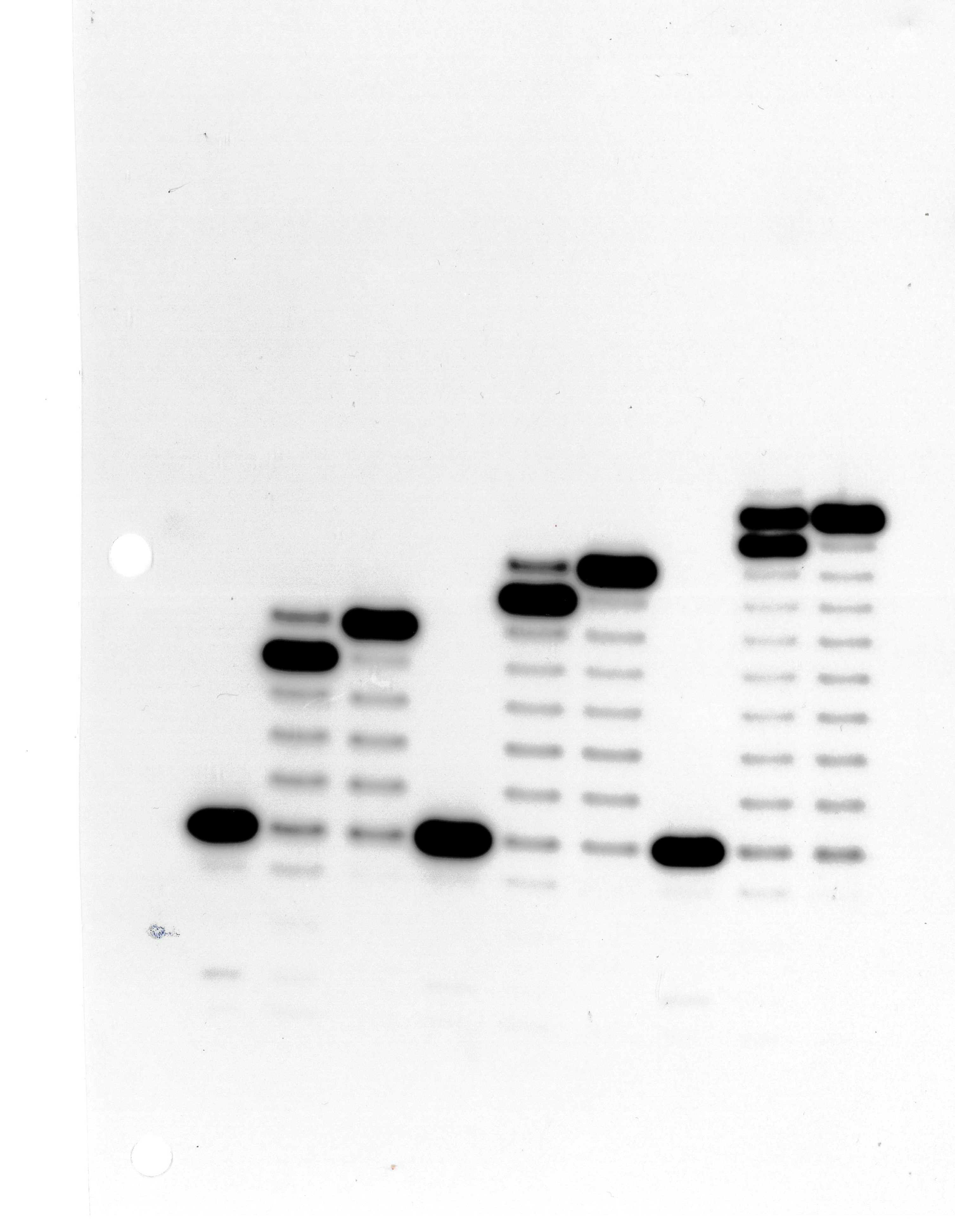

Supplement: Figure 4—figure supplement 1—source data 1. [file elife-83094-fig4-figsupp1-data1.zip › Fig4-fig supp 1/Fig4-fig supp 1B/Fig 4-fig supp 1B no label.tif]

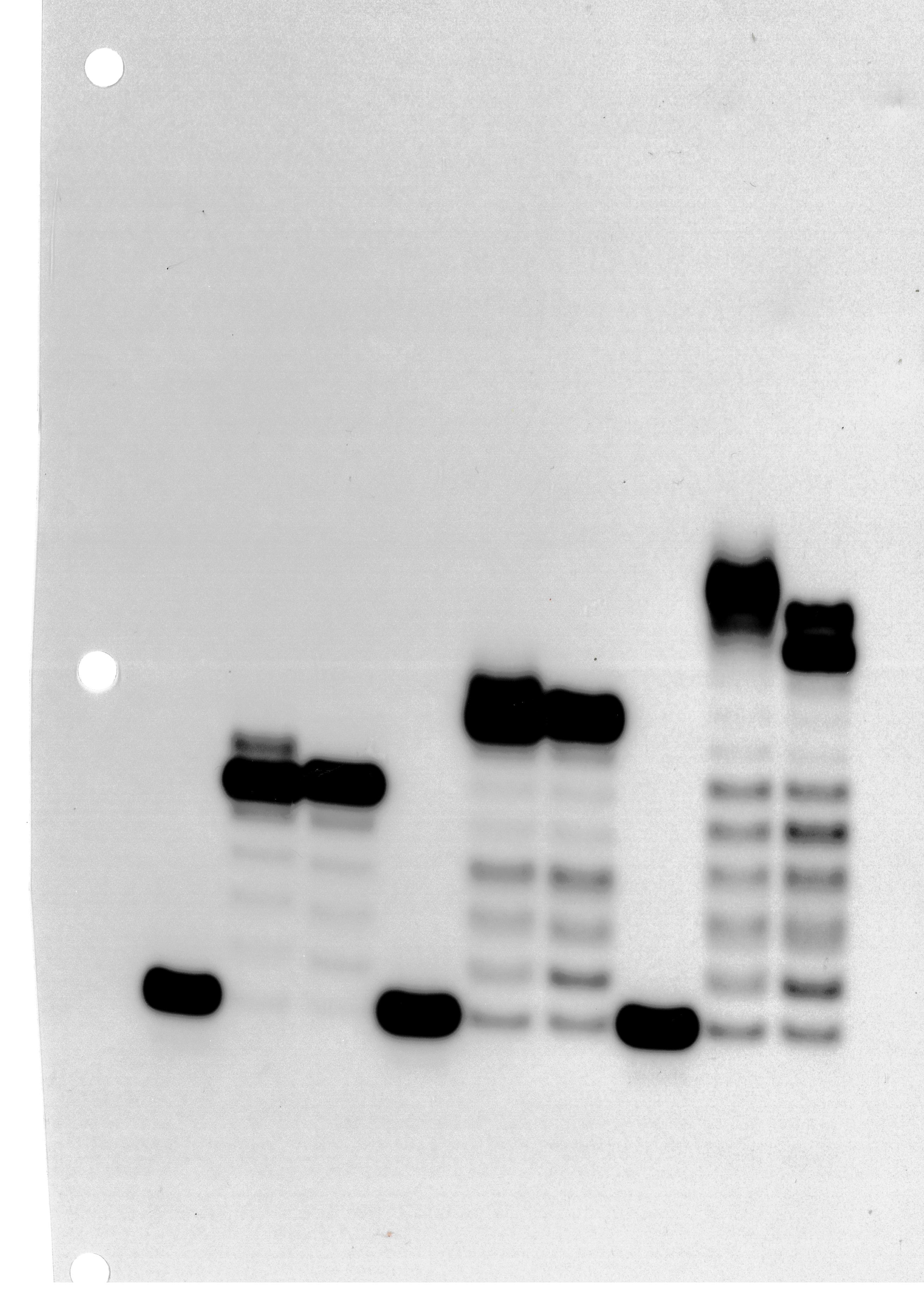

Supplement: Figure 4—figure supplement 1—source data 1. [file elife-83094-fig4-figsupp1-data1.zip › Fig4-fig supp 1/Fig4-fig supp 1C/Fig 4-fig supp 1C no label.tif]

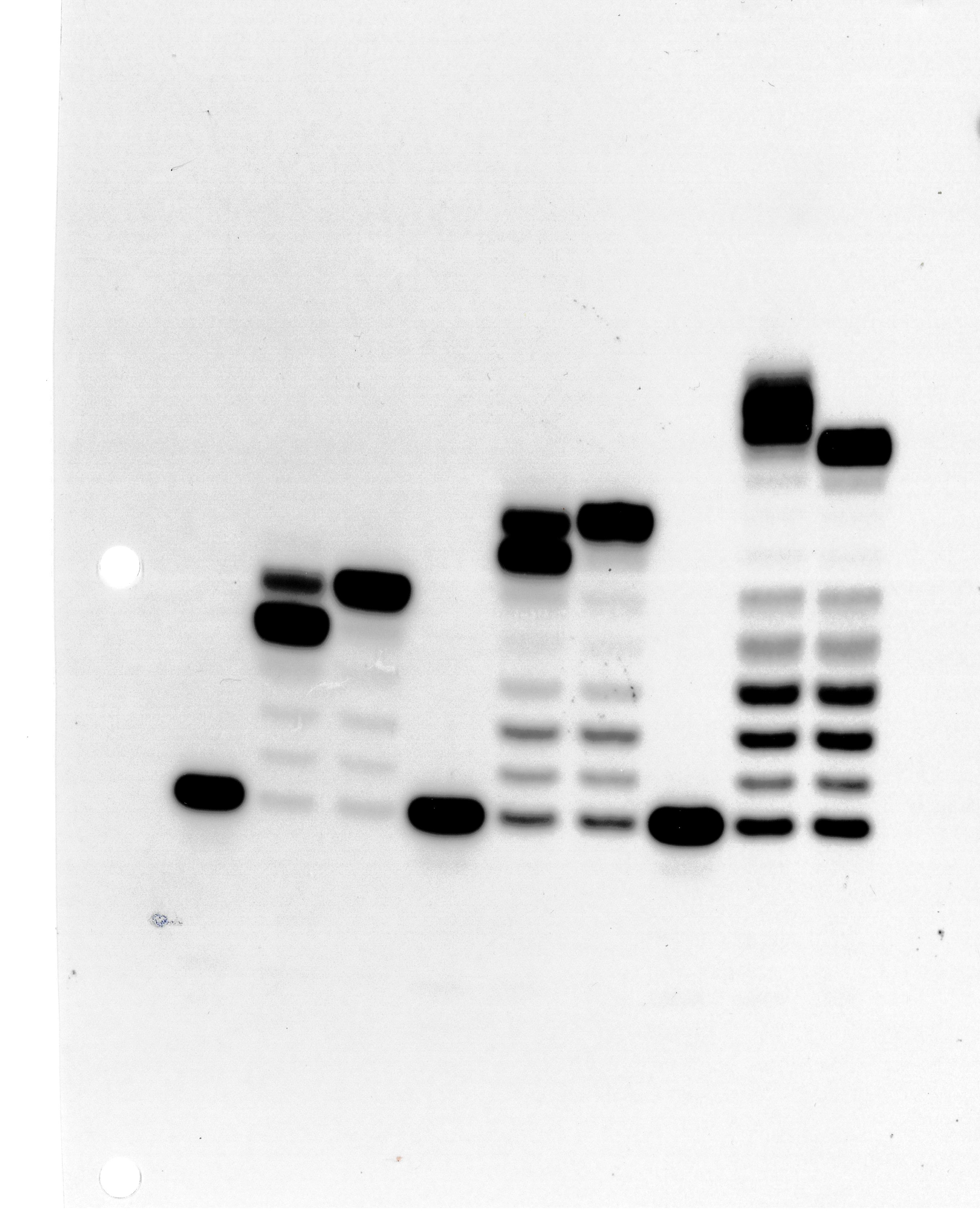

Supplement: Figure 4—figure supplement 1—source data 1. [file elife-83094-fig4-figsupp1-data1.zip › Fig4-fig supp 1/Fig4-fig supp 1D/Fig 4-fig supp 1D no label.tif]

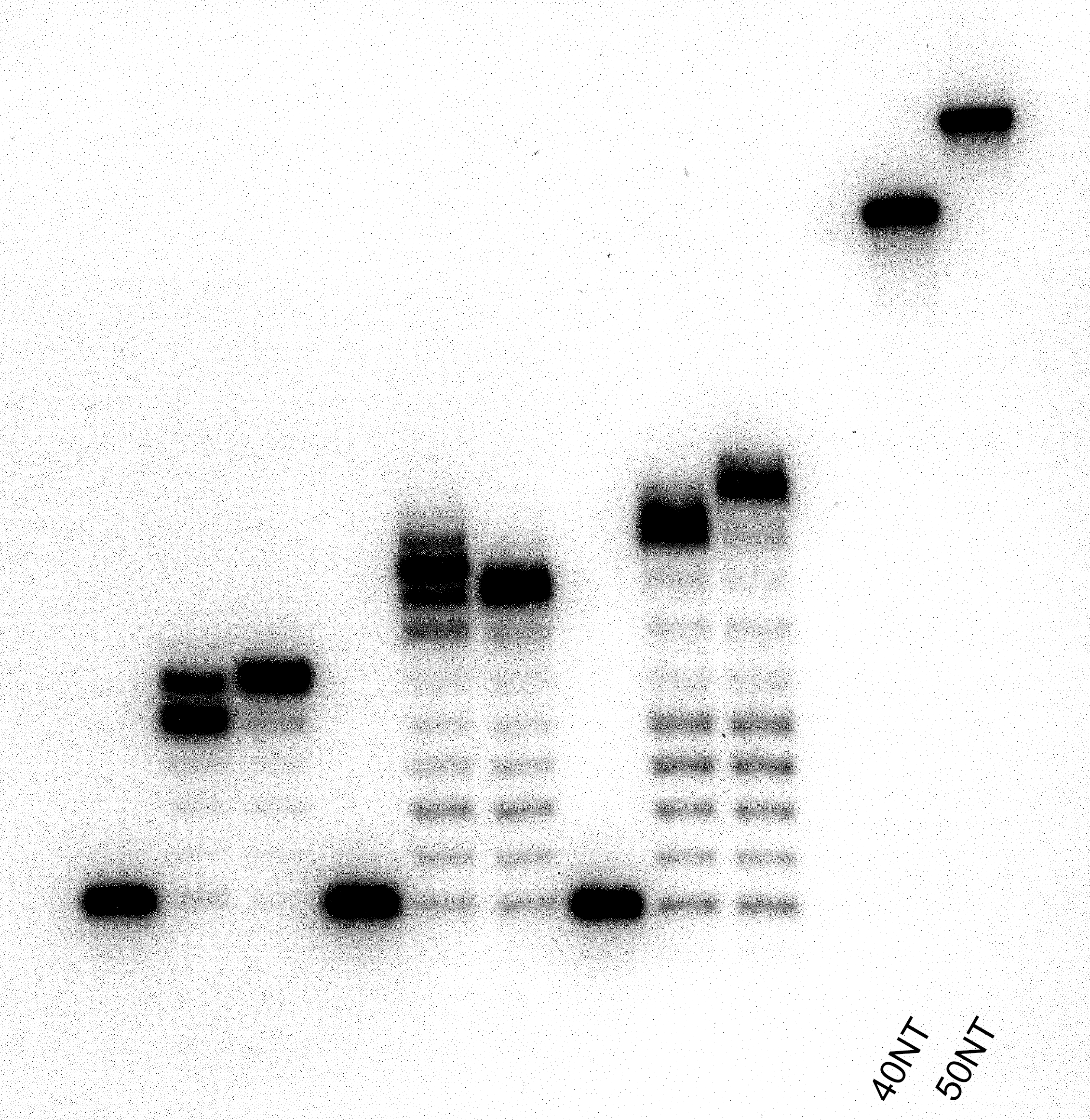

Supplement: Figure 5—source data 1. [file elife-83094-fig5-data1.zip › Fig 5/5I/Fig 5I labels.tif]

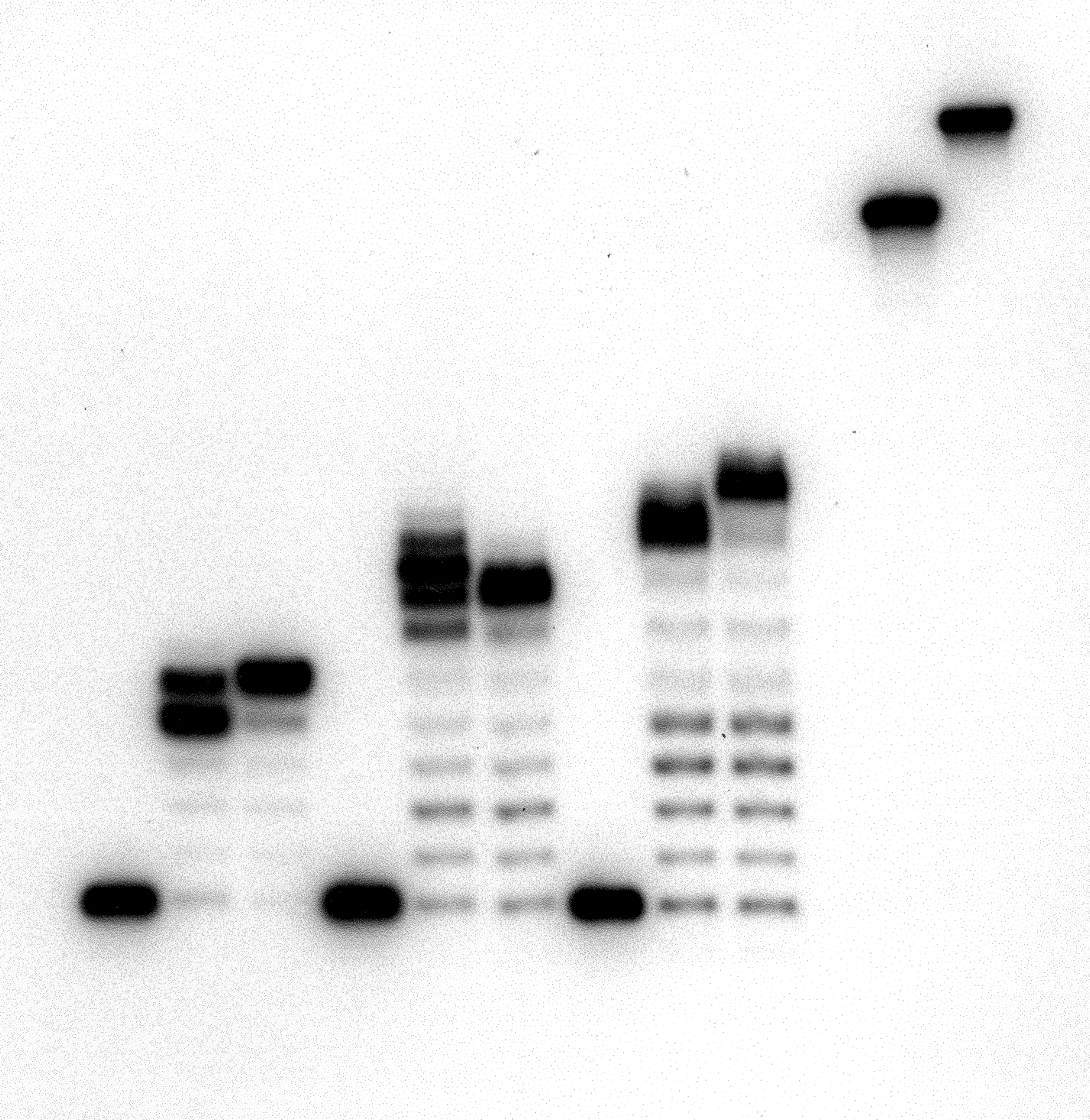

Supplement: Figure 5—source data 1. [file elife-83094-fig5-data1.zip › Fig 5/5I/Fig 5I no label.tif]

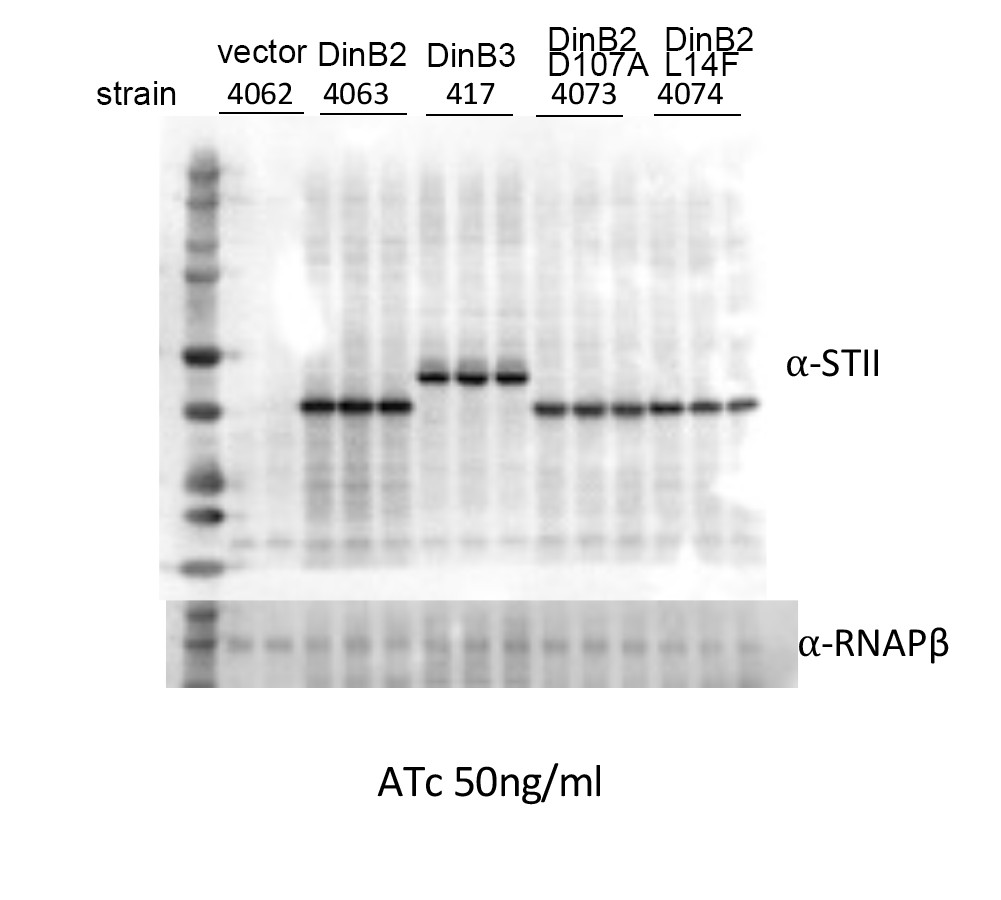

Supplement: Figure 5—source data 1. [file elife-83094-fig5-data1.zip › Fig 5/5A/Fig 5A labels.tif]

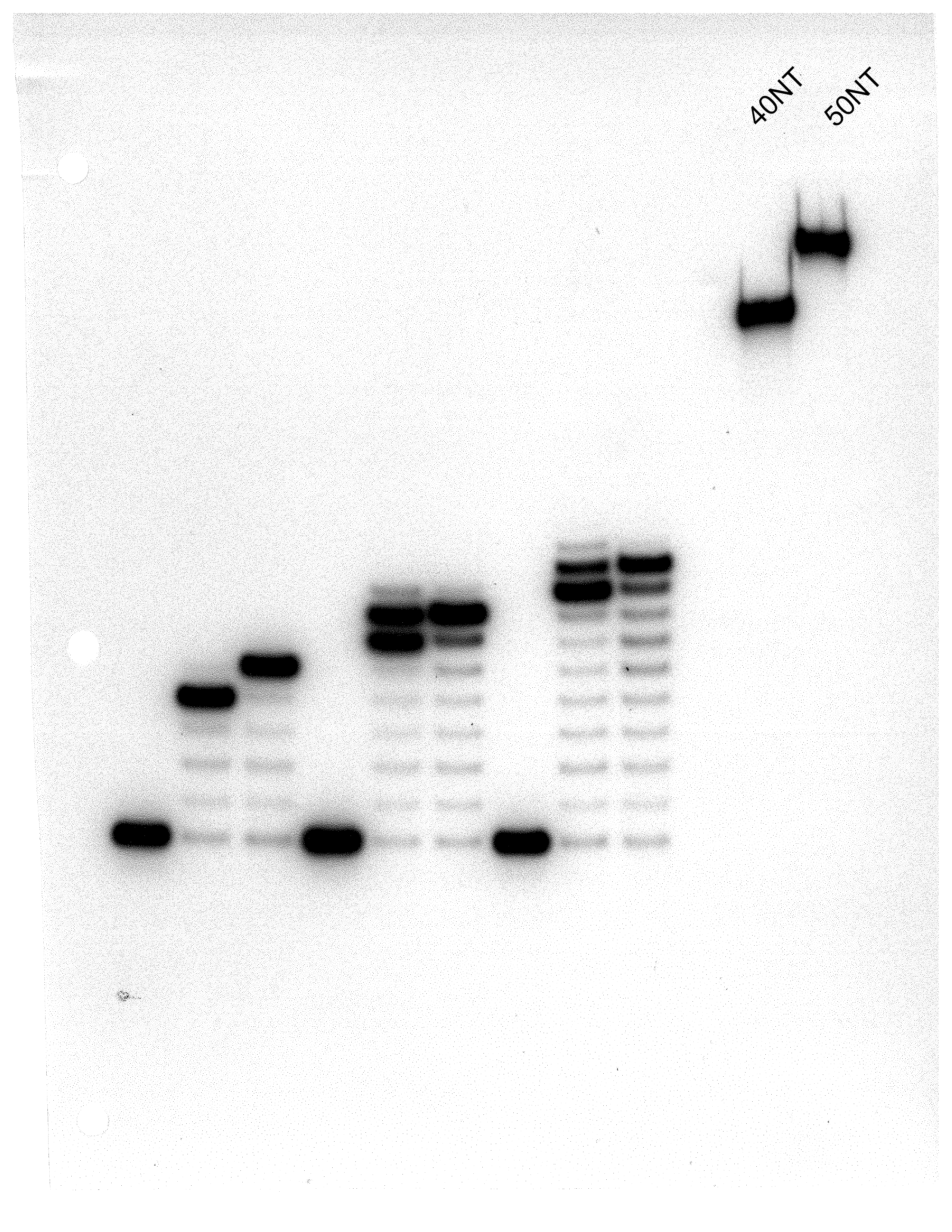

Supplement: Figure 5—source data 1. [file elife-83094-fig5-data1.zip › Fig 5/5H/Fig 5H label.tif]

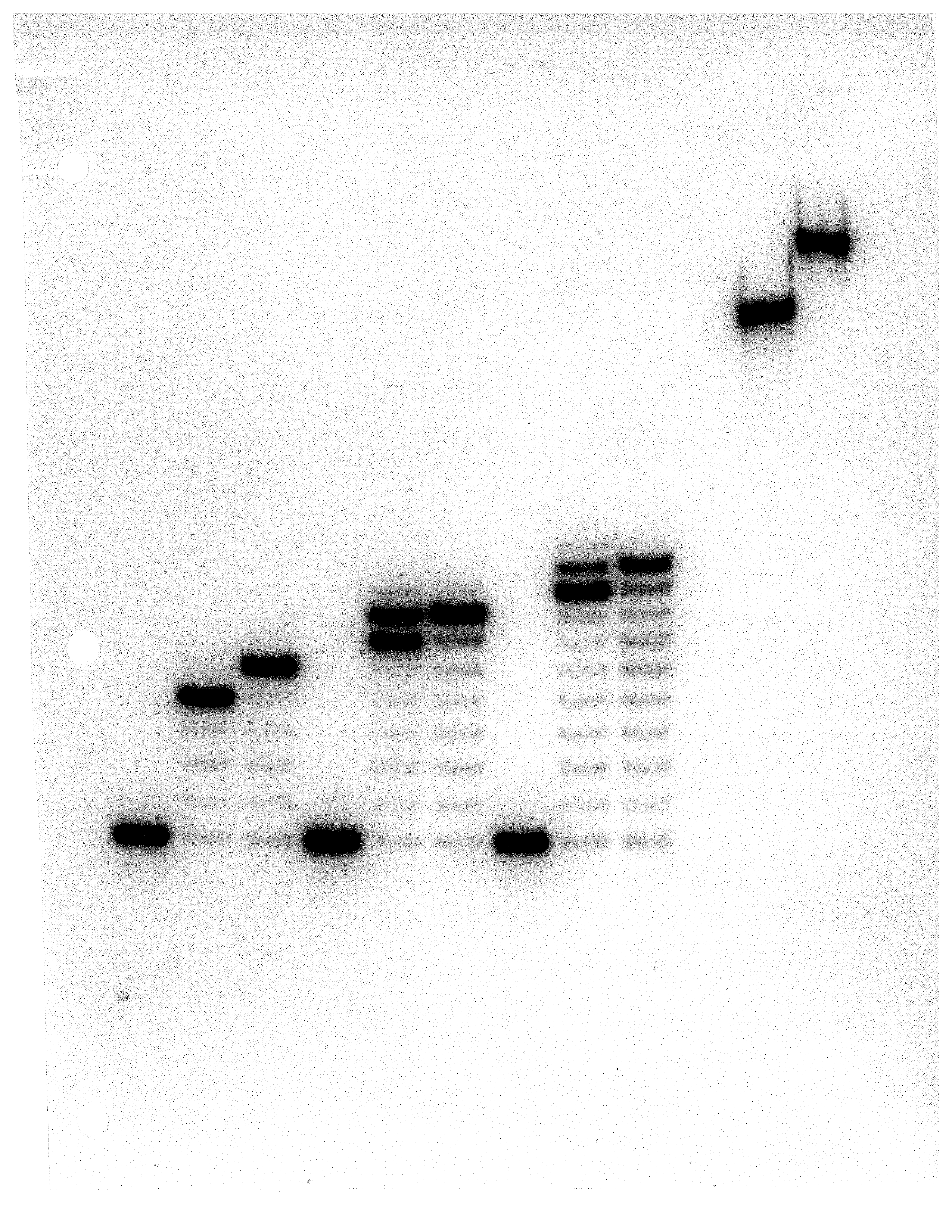

Supplement: Figure 5—source data 1. [file elife-83094-fig5-data1.zip › Fig 5/5H/Fig 5H no label.tif]

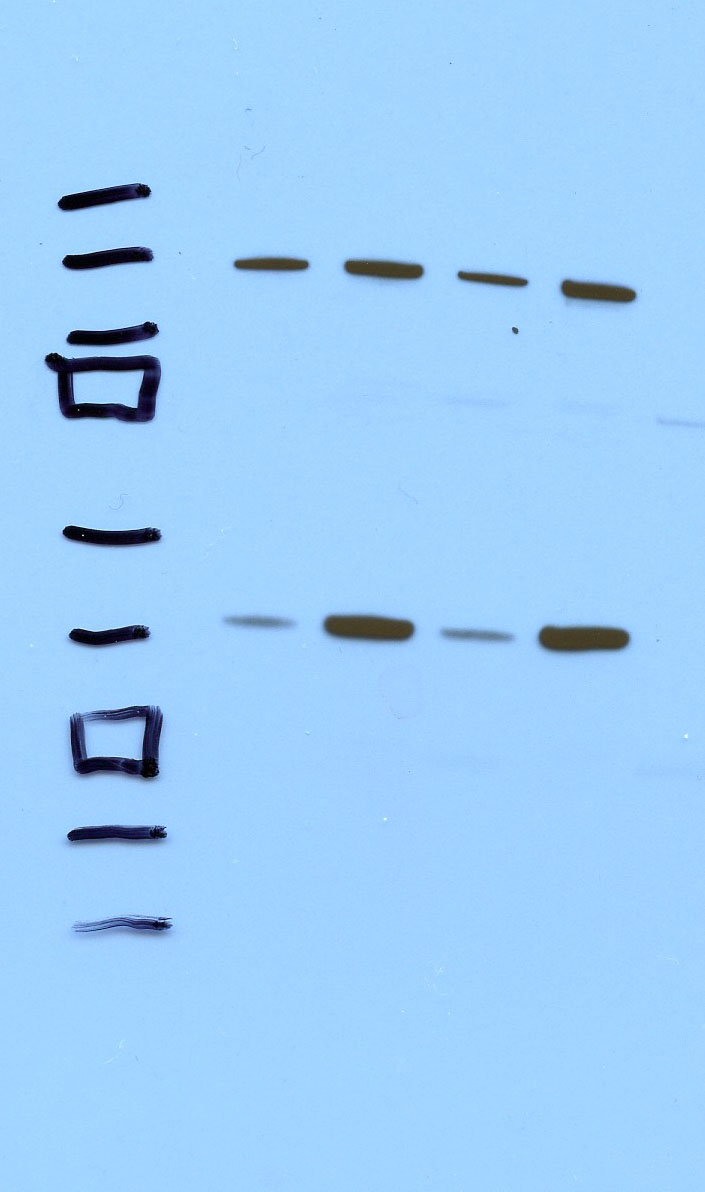

Supplement: Figure 5—source data 1. [file elife-83094-fig5-data1.zip › Fig 5/5D/Fig 5D no label.tif]

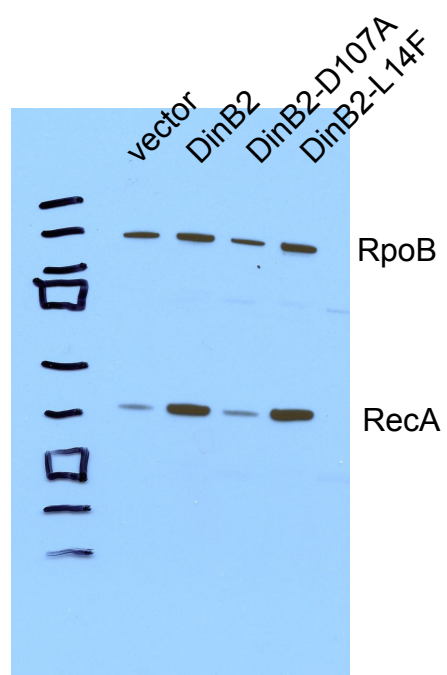

Supplement: Figure 5—source data 1. [file elife-83094-fig5-data1.zip › Fig 5/5D/Fig 5D label.pdf]

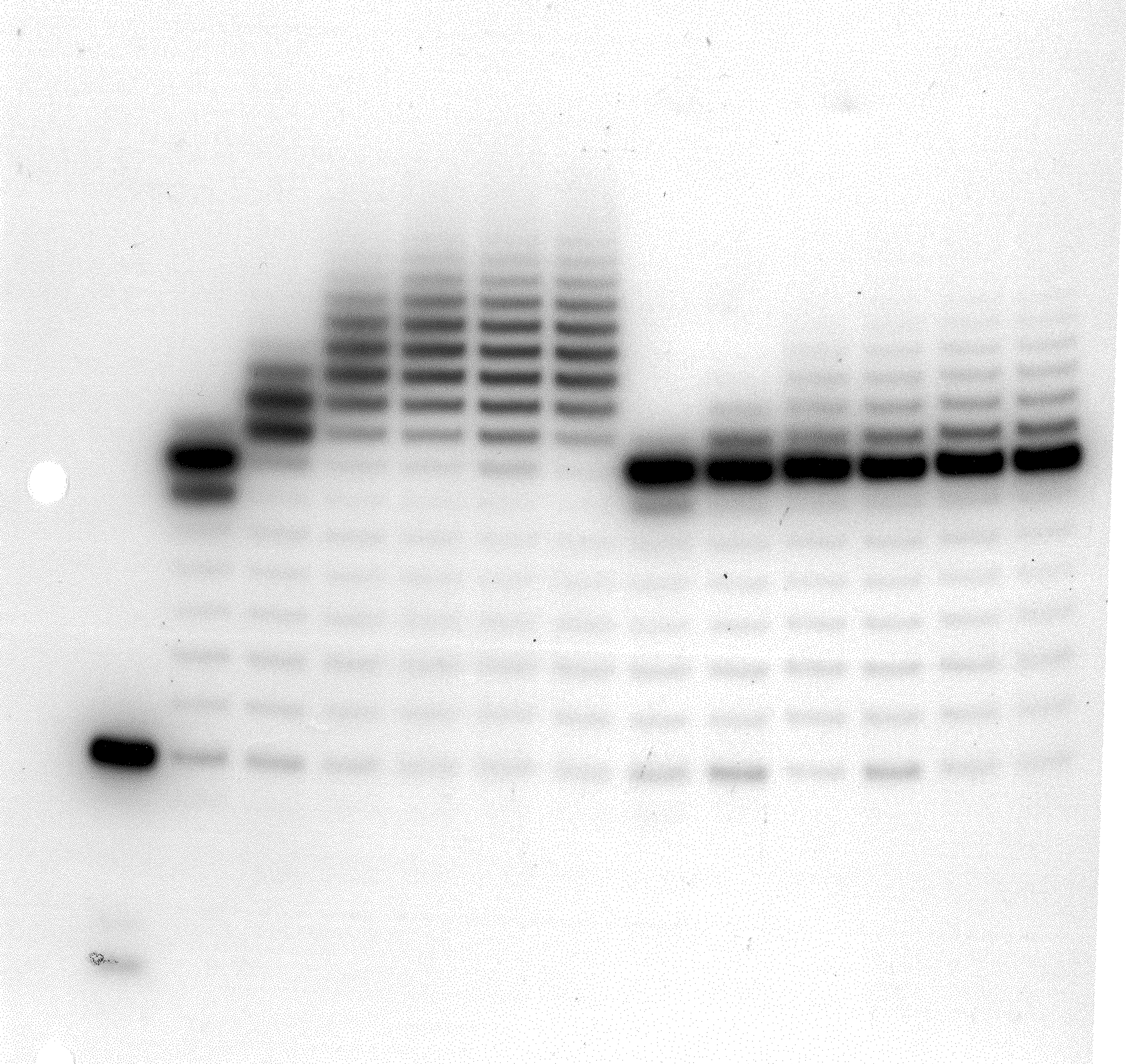

Supplement: Figure 6—source data 1. [file elife-83094-fig6-data1.zip › Fig 6/6A/Fig 6A no label.tiff]

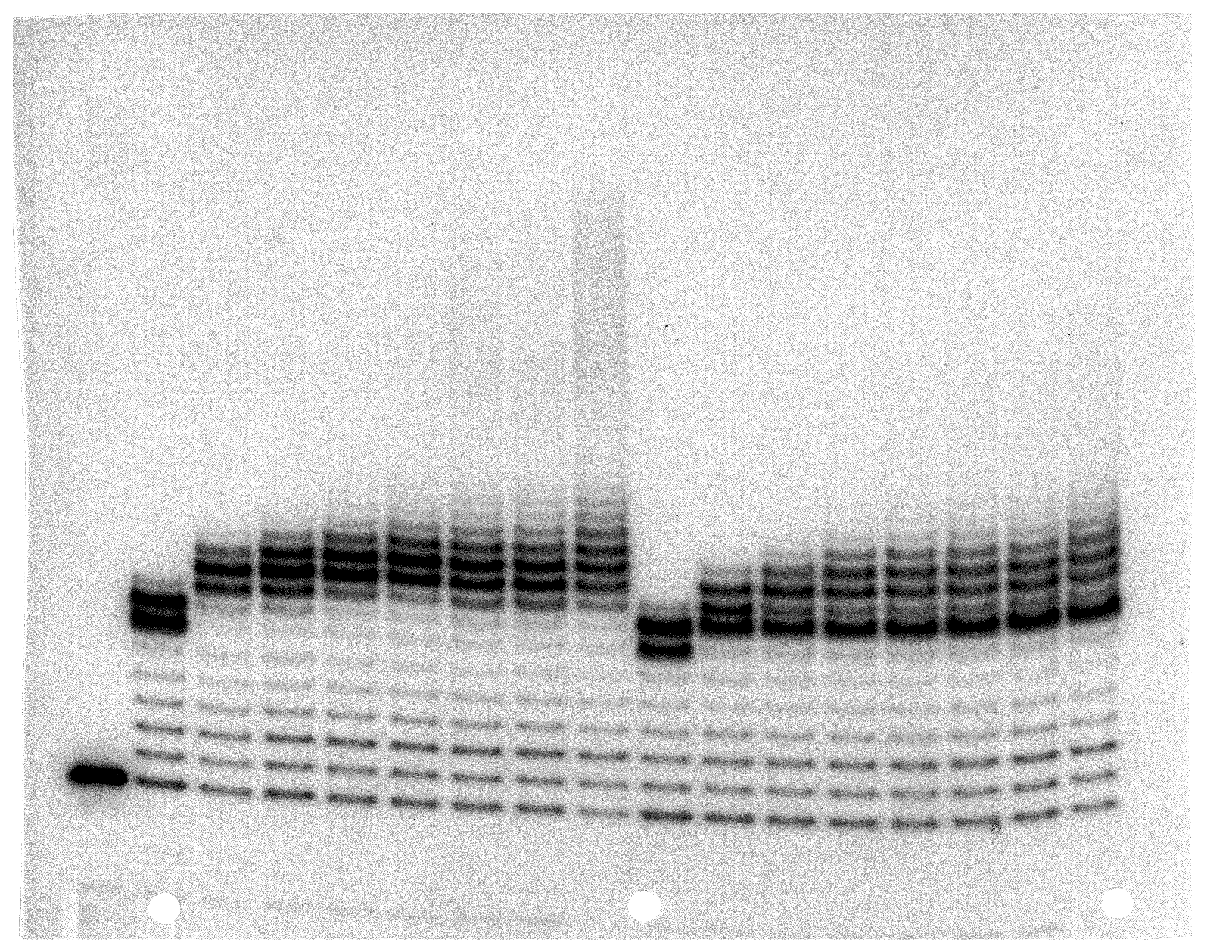

Supplement: Figure 6—source data 1. [file elife-83094-fig6-data1.zip › Fig 6/6B/Fig 6B no label.tiff]

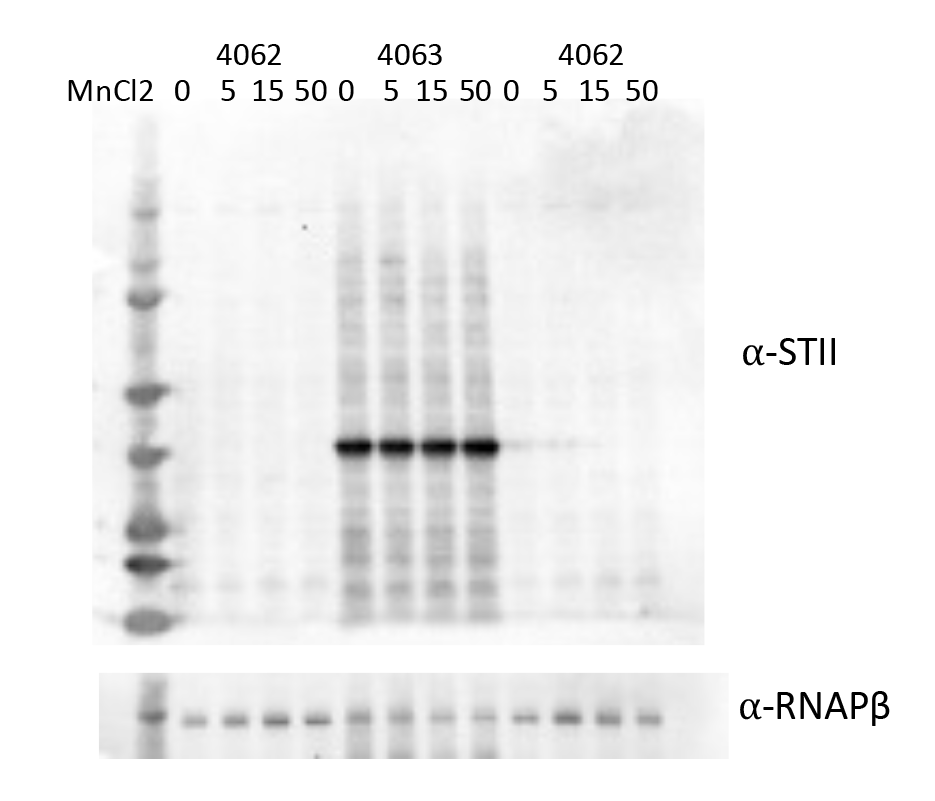

Supplement: Figure 6—source data 1. [file elife-83094-fig6-data1.zip › Fig 6/6C/Fig 6C labels.tif]

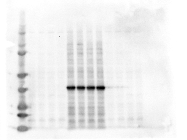

Supplement: Figure 6—source data 1. [file elife-83094-fig6-data1.zip › Fig 6/6C/Fig 6C no label.tif]
